# Folic acid and melatonin ameliorate carbon tetrachloride-induced hepatic injury, oxidative stress and inflammation in rats

**DOI:** 10.1186/1743-7075-10-20

**Published:** 2013-02-03

**Authors:** Hossam Ebaid, Samir AE Bashandy, Ibrahim M Alhazza, Ahmed Rady, Sultan El-Shehry

**Affiliations:** 1 Department of Zoology, College of Science, King Saud University, KSA, P.O.Box, 2455, Riyadh, 11451, Saudi Arabia; 2 Department of Zoology, College of Science, El-Minia University, El-Minia, Egypt; 3 Department of Pharmacology, National Research Center, Cairo, Egypt; 4 Fetal Programming of Disease Research Chair, King Saud University, KSA, Riyadh, Saudi Arabia

**Keywords:** Hepatic injury-markers, Melatonin, Folic acid, Akt1, Oxidative stress, Anti-inflammatory effects

## Abstract

This study investigated the protective effects of melatonin and folic acid against carbon tetrachloride (CCl4)-induced hepatic injury in rats. Oxidative stress, liver function, liver histopathology and serum lipid levels were evaluated. The levels of protein kinase B (Akt1), interferon gamma (IFN-γ), programmed cell death-receptor (Fas) and Tumor necrosis factor-alpha (TNF-α) mRNA expression were analyzed. CCl4 significantly elevated the levels of lipid peroxidation (MDA), cholesterol, LDL, triglycerides, bilirubin and urea. In addition, CCl4 was found to significantly suppress the activity of both catalase and glutathione (GSH) and decrease the levels of serum total protein and HDL-cholesterol. All of these parameters were restored to their normal levels by treatment with melatonin, folic acid or their combination. An improvement of the general hepatic architecture was observed in rats that were treated with the combination of melatonin and folic acid along with CCl4. Furthermore, the CCl4-induced upregulation of TNF-α and Fas mRNA expression was significantly restored by the three treatments. Melatonin, folic acid or their combination also restored the baseline levels of IFN-γ and Akt1 mRNA expression. The combination of melatonin and folic acid exhibited ability to reduce the markers of liver injury induced by CCl4 and restore the oxidative stability, the level of inflammatory cytokines, the lipid profile and the cell survival Akt1 signals.

## Introduction

Carbon tetrachloride (CCl4) is a well-known compound for the production of chemical hepatic injury [1] mediated by metabolites that react with antioxidant enzymes, such as glutathione (GSH), catalase and superoxide dismutase, [2] and increase the level of inflammatory cytokines. Antioxidants exhibit a strong protection against CCl4-induced hepatic toxicity [3,4]. Previous studies have shown that melatonin and folic acid are very potent antioxidants that scavenge reactive oxygen species (ROS) [5]. The release of melatonin, a pineal gland hormone [6], is mainly implicated in the regulation of the sleep/wake cycle and the hormonal regulation of sexual development [7]. Melatonin has been found to be more effective in the protection against oxidative damage than other antioxidants, including vitamin E, glutathione and mannitol [8]. However, the circulating levels of melatonin following oral administration can increase both the mRNA levels and the activities of antioxidant enzymes [9]. The administration of melatonin to aged mice was able to inhibit increase the relative expression of pancreatic genes that are involved in inflammation, oxidative stress and apoptosis [10] and decrease the mRNA expression of Tumor necrosis factor-alpha (TNF-α), interleukin-1 β (IL-1β), nuclear factor (NF)-κB and transcriptional repressor (NKAP) [11]. Melatonin also reduces cytokine levels in surgical neonates [12]. Melatonin prevents hemorrhagic shock-induced liver injury in rats through an Akt-dependent HO-1 pathway [13].

Epidemiological studies have shown that folic acid supplementation can reduce the risk of cardiovascular and hematological diseases [14], neurological and neuropsychiatric disorders [15], neural tube defects [16] and several types of cancer, including cervical, lung, brain, pancreatic, colorectal and breast cancer [17]. The antioxidant activity of folic acid is thought to be involved in these effects of folic acid on health [18]. In fact, folic acid has been reported to have an antioxidant effect against ROS and an alleviating role in hyperhomocysteinemia and its associated endothelial dysfunction [19]. Moreover, the anti-inflammatory effect of folic acid is manifested by a decrease in the levels of interleukin and C-reactive proteins [20].

The combination of melatonin and folic acid improves oocyte quality and thus the pregnancy outcome in women with a history of low oocyte quality [21]. In particular, because melatonin and folic acid are very potent antioxidants in the scavenging of ROS, their effects on CCl4-induced hepatic toxicity were investigated in this study. However, no reports are currently available on the protective effect of folic acid alone or in combination with melatonin against the oxidative stress that occurs from CCl4- induced hepatic injury, which is the subject of this study. Folates function as cofactors in the transfer and utilization of carbon groups and therefore play a key role in the biosynthesis of purines and pyrimidines and the regeneration of methionine. The re-methylation of homocysteine to form methionine requires folate, which alters the secretion of melatonin in rats [22]. Therefore, we hypothesized that the combination of folic acid and melatonin would prove useful in the prevention of various liver injuries induced by oxidative stress.

## Materials and methods

### Animals and experimental design

A total of 56 male Wistar rats (*Rattus norvegicus*) weighing 150–170 g (20 ± 1 weeks) was obtained from the College of Pharmacy, King Saud University. Throughout the experiment, the animals were housed in polypropylene cages, one for each group of eight animals. They were allowed to acclimatize to the laboratory environment for seven days before the beginning of the experiment. The animals were maintained at 18-22°C, kept on a 12:12 h light:dark cycle and provided with food and water *ad libitum*. All animal procedures were conducted in accordance with the standards set forth in the guidelines for the care and use of experimental animals by the Committee for the Purpose of Control and Supervision of Experiments on Animals and the National Institutes of Health. The study protocol was approved by the Animal Ethics Committee of the Zoology Department in the College of Science at King Saud University.

The rats were divided into seven groups, each of which contained eight rats. Group 1 served as the control and only received the vehicle. Group 2 received a single dose of 1 ml/kg CCl4 in liquid paraffin (1:1 volume) through an intraperitoneal (IP) injection [23] (inoculated dose was 0.1 ml). Groups 3 and 4 were pretreated with a daily dose of 2.5 mg/kg folic acid and 10 mg/kg melatonin S.C, respectively [24], for 3 weeks before receiving the CCl4 challenge. Group 5 received the same doses of folic acid as group 3; similarly, Group 6 was treated with the same melatonin treatment protocol as group 4. Group 7 was pre-treated with a combination of folic acid and melatonin for 3 weeks before CCl4 challenge. The samples were collected 2 days after the injection of CCl4.

### Chemicals

The chemicals 5,5-dithiobis-2-nitrobenzoic acid (DTNB), dihydrogen phosphate, trichloroacetic acid, carbon tetrachloride, and thiobarbituric acid were purchased from Merk Company, Darmstadt, Germany. Melatonin was obtained from MPBiomedicals, LLC, Llkirch, France. Folic acid was purchased from Sigma Chemical Company, Louis, MO, USA.

### Blood and liver samples

Two days after treatment, the animals from all groups were autopsied under light ether anesthesia. The blood was drawn from the animals by puncturing the retro-orbital venous sinus with capillary tubes until killing. The blood was collected in heparin-coated centrifuge tubes, centrifuged at 200 × *g* for 10 minutes, then plasma was separated in Eppendorf tubes and stored at −30°C. Whole blood was used for the determination of the level of hydroperoxide, whereas separated plasma was used to determine the level of liver enzymes, the amount of total protein and the lipid profile.

The liver was removed, washes with saline and cut into two parts, one part was used for the histological study and the other part was used for the assessment of lipid peroxidation (MDA), GSH and catalase. The hepatic tissues were homogenized (Automated homogenizer, IKA, T25D, Germany) in 10 mM KCl in 1.15% phosphate buffer and ethylenediamine tetraacetic acid (EDTA; pH 7.4) and centrifuged at 5000 × g for 10 min. The supernatant was used to assay the level of thiobarbituric acid reactive substances (TBARS) and to estimate the amount of GSH and catalase.

### Biochemical analysis

### Estimation of lipid peroxides

The blood hydroperoxide level was evaluated using the free radical analytical system (Iran, Parma, Italy), which is a colorimetric test that takes advantage of the ability of hydroperoxides to generate free radicals after reacting with some transitional metals. In this test, a colored complex appears when buffered chromogenic substances are added to a solution that contains hydroperixodes. The amount of complex that was formed can then be measured by a spectrophotometer. The lipid peroxidation level, or the amount of TBARS in the liver, was measured by a method described by Ohkawa, et al. [25]. The liver tissue was homogenized in ice-cold 0.15 M HCl (10%) and the absorbance was read at 532 nm. Using 1,1,3,3-tetramethoxypropane as the standard, the absorbance was used to determine the concentration of TBARS, which was expressed as nm of MDA per mg protein.

### Assay of hepatic reduced glutathione

The reduced form of glutathione was determined using DTNB as the coloring reagent and following the method described by Moron et al. [26]. The absorbance was read at 412 nm using a spectrophotometer and the GSH concentration was calculated from a standard curve.

### Determination of hepatic catalase

The level of catalase activity was estimated in the liver homogenate by the method described by Aebi [27]. The specific activity of catalase is expressed in units of moles of H2O2 consumed/min/mg of protein. The difference in the absorbance at 240 nm per unit time was used to determine the catalase activity.

### Liver function tests and lipid profile

The levels of AST, ALT, ALP, bilirubin, LDH, cholesterol, triglycerides, LDL, HDL, protein, and urea were measured in the plasma samples obtained from all the groups. The measurements were performed in accordance with the manufacturer protocols of the Bio Merieux kits, France. The amounts of AST, ALT and ALP were determined kinetically, whereas the other proteins were evaluated by colorimetry. The intensity of coloration was measured using the UV/Visible-Model-80-2106-00 spectrophotometer, Pharmacia Biotech, Cambridge, England.

### Histological study

The liver sample from each animal was processed using light microscopy. The tissue sections were fixed in 10% neutral buffered formalin and embedded in paraffin. The paraffin sections were then stained with hematoxylin-eosin (H&E). Mallory Trichrome was used for detecting the collagen deposition in the hepatic tissue. The degree of liver damage was examined blindly using a Leica DMRB/E light microscope (Heerbrugg, Switzerland).

### RNA extraction and cDNA synthesis

The total RNA from the liver tissue homogenates was isolated using TRIzol reagent (Invitrogen®). The isolation was performed according to the manufacturer’s instructions and quantified by measuring the absorbance at 260 nm. The cDNA synthesis was performed using the High-Capacity cDNA reverse transcription kit (Applied Biosystems®) according to the manufacturer’s instructions. A total of 1.5 μg of total RNA from each sample was added to a mixture of 2.0 μl of 10x reverse transcriptase buffer, 0.8 μl of 25x dNTP mix (100 mM), 2.0 μl of 10x reverse transcriptase random primers, 1.0 μl of MultiScribe reverse transcriptase, and 3.2 μl of nuclease-free water. The final reaction mixture was kept at 25°C for 10 min, heated to 37°C for 120 min, heated to 85°C for 5 s, and then cooled to 4°C.

### Quantification of mRNA expression by real-time polymerase chain reaction

The quantitative analysis of the level of mRNA expression of the target genes was achieved by RT-PCR. The cDNA from the above preparation was subjected to PCR amplification using 96-well optical reaction plates in the ABI Prism 7500 System (Applied Biosystems®). The 25-μl reaction mixture in each well contained 0.1 μl of 10 μM forward primer and 0.1 μl of 10 μM reverse primer for a final concentration of 40 μM of each primer, 12.5 μl of SYBR Green Universal Mastermix (Applied Biosystems®), 11.05 μl of nuclease-free water, and 1.25 μl of the cDNA sample. The primers used in the current study were chosen from the PubMed database (pubmed.com) (Table 1). The RT-PCR data were analyzed using the relative gene expression method, as described by the Applied Biosystems® User Bulletin No. 2. The data are therefore presented as the fold change in gene expression normalized to the endogenous reference gene and relative to a calibration gene.

**Table 1:** Sequences of primer and probes of quantitative RT-PCR

**Primer**	**Sequence**
Fas	F: CTGCCTCTGGTGCTTGCTGGC
	R: ACCCCACCCCCTTCTCCCAATTC
AKt1	F: ACGCCGCCTGATCAAGTTCTCC
	R: TGACGGACAGCGGGAGAGGG
IFN-γ	F: TCTGGGCTTCTCCTCCTGCGG
	R: GGCGCTGGACCTGTGGGTTG
TNF-α	F: GCGGAGTCCGGGCAGGTCTA
	R: GGGGGCTGGCTCTGTGAGGA

### Statistical analysis

The statistical analysis was performed using the MINITAB software (MINITAB, State College, PA, Version 13.1, 2002). The data from the experiments were tested for normality using the Anderson Darling test and for variance homogeneity prior to any further statistical analysis. The data were normally distributed with homogeneous variances. Thus, the one-way ANOVA statistical measure was used to determine overall effect of each treatment. This measure was supplemented by individual comparison between the different treatments using Tukey’s method for pairwise comparisons. The results were expressed as arithmetic mean (M) ± standard deviation (SD). Only statistically significant differences, with P < 0.05, that were found between a treatment group and the control and between a treatment group and the CCl_4_ group were considered.

## Results

### Effects of melatonin and folic acid on CCl4-induced oxidative stress

The results revealed that CCl4 significantly elevated both the concentration of hydroperoxide and the amount of lipid peroxidation. Melatonin, folic acid and their combination were found to significantly reduce this high concentration of hydroperoxide, although it was still significantly higher than that of the control group. Interestingly, the amount of lipid peroxidation was restored to the normal baseline concentration observed in the control group by these three treatments (Table 2). We showed that glutathione, the most important anti-oxidant enzyme, was significantly decreased by CCl4 challenge. However, the concentration of glutathione in animals treated with melatonin, folic acid and their synergic combination was four to six-fold higher (*p* < 0.05) than the concentration of glutathione measured in the CCl4-treated group. Furthermore, these three treatments restored the concentration of catalase activity to values close to that observed in the control group (Table 2).

**Table 2:** Concentrations of oxidative stress parameters and relative mRNA expression of TNF-α, AKT1, Fas and IFN-γ induced in the different groups

**Parameters**	**Control group**	**CCL4**	**Me**	**CCL4+Me**	**Fo**	**CCL4+Fo**	**CCL4+Me + Fo**
Blood Hydroperoxide (mg/100 ml)	24 ± 5.0	44.5* ± 6.2	21.5 ± 4.3	32.1* ± 4.2	21.8 ± 3.0	30.6 ± 6.3	27 ± 5.0
Hepatic MDA (nmol/mg protein)	3 ± 0.7	5.6* ± 0.9	2.1 ± 0.8	4* ± 0.7	2.4 ± 0.7	4.4* ± 0.9	2.1 ± 0.6
Hepatic GSH (μmol/g liver tissue)	12.2 ± 1.8	5* ± 1.8	25.7* ± 7.8	14.7 ± 5.0	28.6* ± 5.8	21.7* ± 5.0	21.2* ± 5.1
Catalase (Unit/mg protein)	14.4 ± 1.8	7.9* ± 1.0	16.3 ± 1.8	11.5 ± 2.8	16 ± 2.7	10* ± 0.9	14 ± 1.5
Relative expression of TNF-α mRNA	1.8 ± 0.8	5.6* ± 0.9	1.6 ± 0.5	3.3* ± 0.7	2.1 ± 0.8	3.2* ± 0.7	1.7 ± 0.5
Relative expression of Akt1 mRNA	5.7 ± 1.1	1.8* ± 0.5	15.2* ± 2.7	5.0 ± 1.3	17* ± 1.8	5.0 ± 1.6	7.0 ± 1.8
Relative expression of Fas mRNA	0.9 ± 0.3	10.1* ± 0.9	0.8 ± 0.45	8.2* ± 1.2	0.78 ± 0.2	5.5* ± 0.2	2.5* ± 0.7
Relative expression of IFN- γ mRNA	18.5 ± 7.2	7.0* ± 2.2	17.5 ± 6.0	8.8* ± 0.9	34.2* ± 4.4	14.6 ± 2.6	20.2 ± 3.0

The results are expressed as M ± SD. * shows a statistically significant difference (P < 0.05) in the pairwise comparison between a treatment group and the control.

### Effect of melatonin and folic on CCl4-induced TNF-α, Akt1, Fas and IFN-γ mRNA expression

The expected CCl4-induced hepatocytotoxic effects were confirmed by the significantly elevated apoptosis receptor Fas and TNF-α mRNA expression levels, but either melatonin, folic acid or their combination down regulated these expression levels to those observed in the control group (Table 2). In addition, these three treatments were found to significantly upregulate the expression of the Akt1 and IFN-γ mRNA compared to the CCl4 group. This restoration of the Akt1 and IFN-γ gene expressions in the animals treated with the combination of melatonin and folic acid was superior to that observed in the animals treated with only one of these compounds.

### Biochemical analysis and Histopathological examination

#### Effects of melatonin and folic acid on CCl4-induced liver enzyme concentrations

The results presented in Table 3 show that CCl4 was found to significantly elevate the levels of ALP, ALT, LHD and AST to concentrations that are two-, ten-, two and a half-, three and a half-, six- and thirty-fold their normal values, respectively. In addition, the combination of melatonin and folic acid was found to significantly restore the concentrations of both ALP and LDH to their baseline concentrations. Although the individual treatments with melatonin or folic acid did not normalize the concentrations of ALT and AST to the values observed in the control group, they did significantly decrease the concentrations of these enzymes compared to that observed in the CCl4-group (Table 3).

**Table 3:** Plasma concentrations of liver enzymes, lipids, total protein, bilirubin and urea from the control and the different groups

**Parameters**	**Control group**	**CCL4**	**Me**	**CCL4+Me**	**Fo**	**CCL4+Fo**	**CCL4+Me + Fo**
ALP (U/L)	232.6 ± 33.5	418.3* ± 67	238.6 ± 62	279.1 ± 34	234.7 ± 43	267.3 ± 61	228 ± 35
ALT (U/L)	27 ± 3.8	369* ± 106	29 ± 3.0	293.5* ± 34	22.7 ± 1.1	205.5* ± 77	156.5* ± 62
AST (U/L)	48.7 ± 4.0	369* ± 91	42.1 ± 4.8	251* ± 48	41 ± 7.2	288.7* ± 112	237.7* ± 71
LDH (U/L)	192.3 ± 63	576* ± 221	165.3 ± 47	317.3 ± 110	178.6 ± 43	363.3 ± 130	337.5 ± 143
Cholesterol (mg/100 ml)	57.7 ± 8.0	87* ± 14	53 ± 6.0	59 ± 9.0	61 ± 5.0	76* ± 14	57 ± 8.6
LDL-C (mg/100 ml)	33 ± 6.6	50* ± 15	37 ± 6.0	38 ± 2.3	36 ± 5.7	44* ± 5.0	36 ± 6.0
HDL (mg/100 ml)	31 ± 5.5	10* ± 4.9	32 ± 5.6	16* ± 7.0	30 ± 5.4	24 ± 7.3	27 ± 6.3
Triglycerides (mg/100 ml)	66 ± 4.3	125* ± 10	51 ± 6.0	82* ± 7.0	65 ± 6.2	70 ± 6.2	66 ± 3.2
Plasma Protein g/100 ml	7.7 ± 0.6	5.5* ± 0.5	7.3 ± 0.9	5.8* ± 0.35	6.7 ± 0.7	5.7* ± 0.7	6.7 ± 0.7
Bilirubin Mg/100 ml	0.246 ± 0.08	0.468 ± 0.17	0.226 ± 0.04	0.324 ± 0.06	0.266 ± 0.06	0.42* ± 0.1	0.355 ± 0.07
Urea Mg/100 ml	40.6 ± 7.3	55.5* ± 10	33.6 ± 5.9	55* ± 9.5	50.8 ± 7.0	48.5 ± 3.0	37.6 ± 5.4

The results are expressed as the M ± SD. * shows a statistically significant difference (*P* < 0.05) in the pairwise comparison between a treatment group and the control.

### Effect of combination of melatonin and folic acid on CCl4 induced lipid profile

The cardiovascular risk indices were significantly elevated as a result of CCl4-induced hepatic injury, and treatment with melatonin and folic acid restored these indices. In addition, we found that CCl4 significantly elevated the concentrations of total cholesterol, LDL cholesterol and triglycerides. However, melatonin, folic acid and the combination of these two treatments significantly restored the concentration of these parameters to that measured in the control group. Interestingly, HDL was found to be significantly reduced by the CCl4 challenge, a decrease that was not observed when the animals received any of the three treatments (Table 3).

### Effects of melatonin and folic acid on CCl4-induced concentration of total protein, bilirubin and urea

The results revealed that CCl4 significantly decreased the concentration of total protein in the serum. Treatment with either melatonin or folic acid individually failed to restore the normal concentration of total protein. However, the synergistic combination of folic acid and melatonin was uniquely able to restore the concentration of total protein to a value that is close to that measured in the control group. Unlike its effect on the total protein concentration, the amounts of bilirubin and urea in the serum of CCl4-challenged rats was two and one and a half-fold higher than the concentration in the serum of control rats. We generally observed that the treatments with melatonin or with the combination of melatonin and folic acid were better able to restore the concentrations of these two parameters than treatment with folic acid (Table 3).

### Effect of combination of melatonin and folic acid on the histology of liver

**Figure 1:**
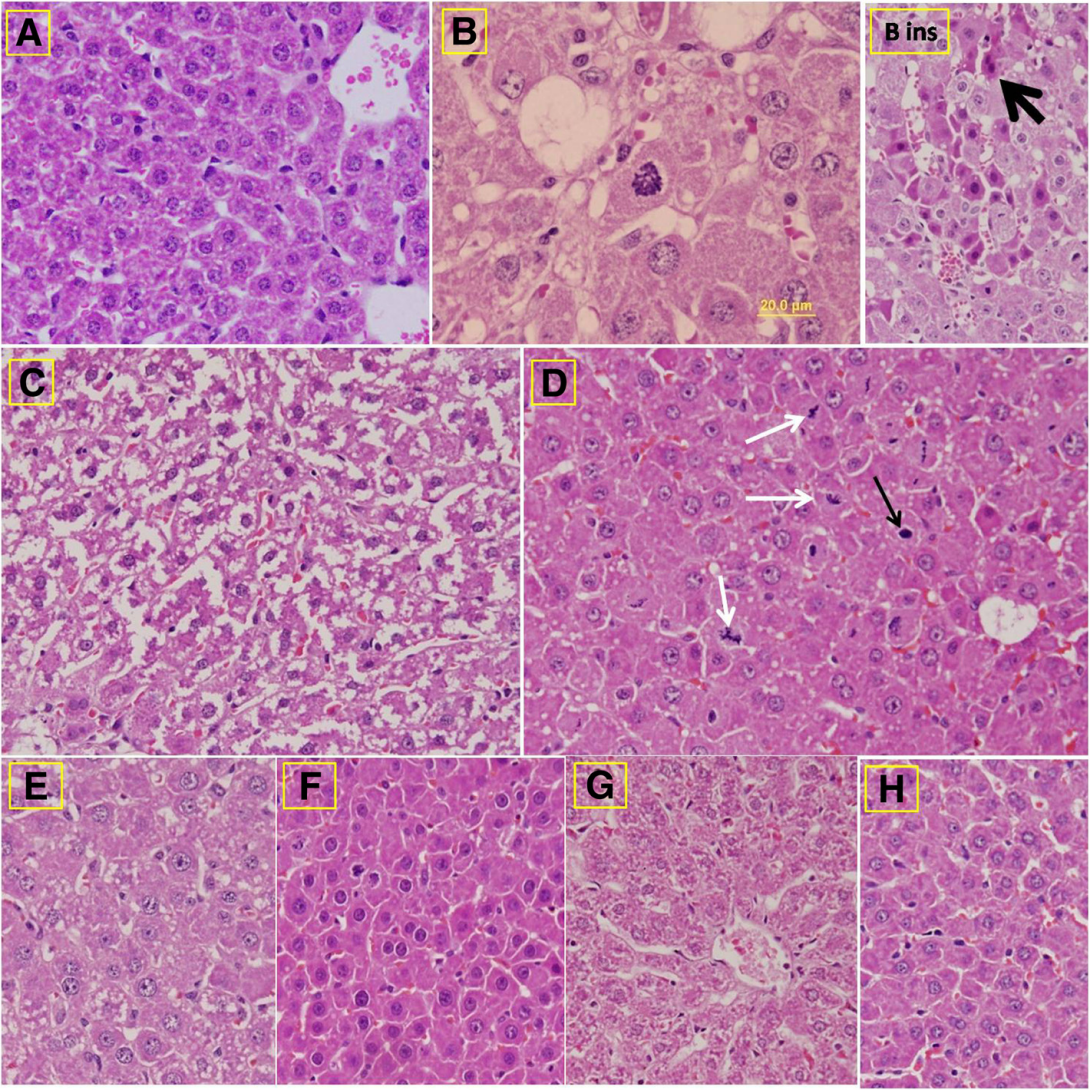
**Photomicrographs for liver sections of the different groups A**: **Photomicrograph of a control liver section showing the normal histological architecture of hepatic tissues (H&E stain, 400x). B**: The histological features of a representative liver section from CCl4-treated rats (the sections from four rats in this group were investigated), which shows the increased number of mitotic figures (arrow) and the clear vacuolation of the hepatocytes (H&E stain, 1000x). Insert: In addition to the mitotic figures, eosinophilic hepatocytes (arrow) were detected in sections from two of the CCl4-treated rats in this group (H&E stain, 400x). **C**: A representative liver section from CCl4 + Me-treated rats (H&E stain, 400x) was characterized by the absence of the mitotic figures. However, vacuolated hepatocytes and abnormal damaged hepatic tissues were still found. **D**: A representative liver section from CCl4 + Fo-treated rats shows many mitotic figures (white arrows), which resemble those detected in the liver sections from CCl4-treated rats. A number of pyknotic nuclei were detected in the sections from this group (H&E stain, 400x). **E**: A representative liver section from CCl4 + Me + Fo-treated rats, which exhibit an improvement of the general hepatic architecture (sections from five rats in this group were investigated) (H&E stain, 400x). **F**: A liver section from the group of CCl4 + Me + Fo-treated rats that shows little improvement (this was observed in the sections from two of the rats) (H&E stain, 400x). **G**: A representative liver section from Fo-treated rats, which shows the general hepatic architecture with a small amount of side effects, such as faintly stained nuclei (H&E stain, 400x). **H**: A representative liver section from Me-treated rats, which shows the general hepatic architecture with narrow hepatic sinusoids and a large number of hepatocytes (H&E stain, 400x).

An increased number of mitotic figures, vacuolated hepatocytes, and eosinophilic hepatocytes characterized the histological sections of the CCl4-challenged group. The liver sections obtained from CCl4-challenged rats that were treated with folic acid showed a large number of mitotic figures, which were not present in the liver sections obtained from CCl4-challenged rats that were treated with melatonin. In addition, the combined treatment of folic acid and melatonin resulted in an improvement in the general hepatic architecture (Figure 1).

**Figure 2:**
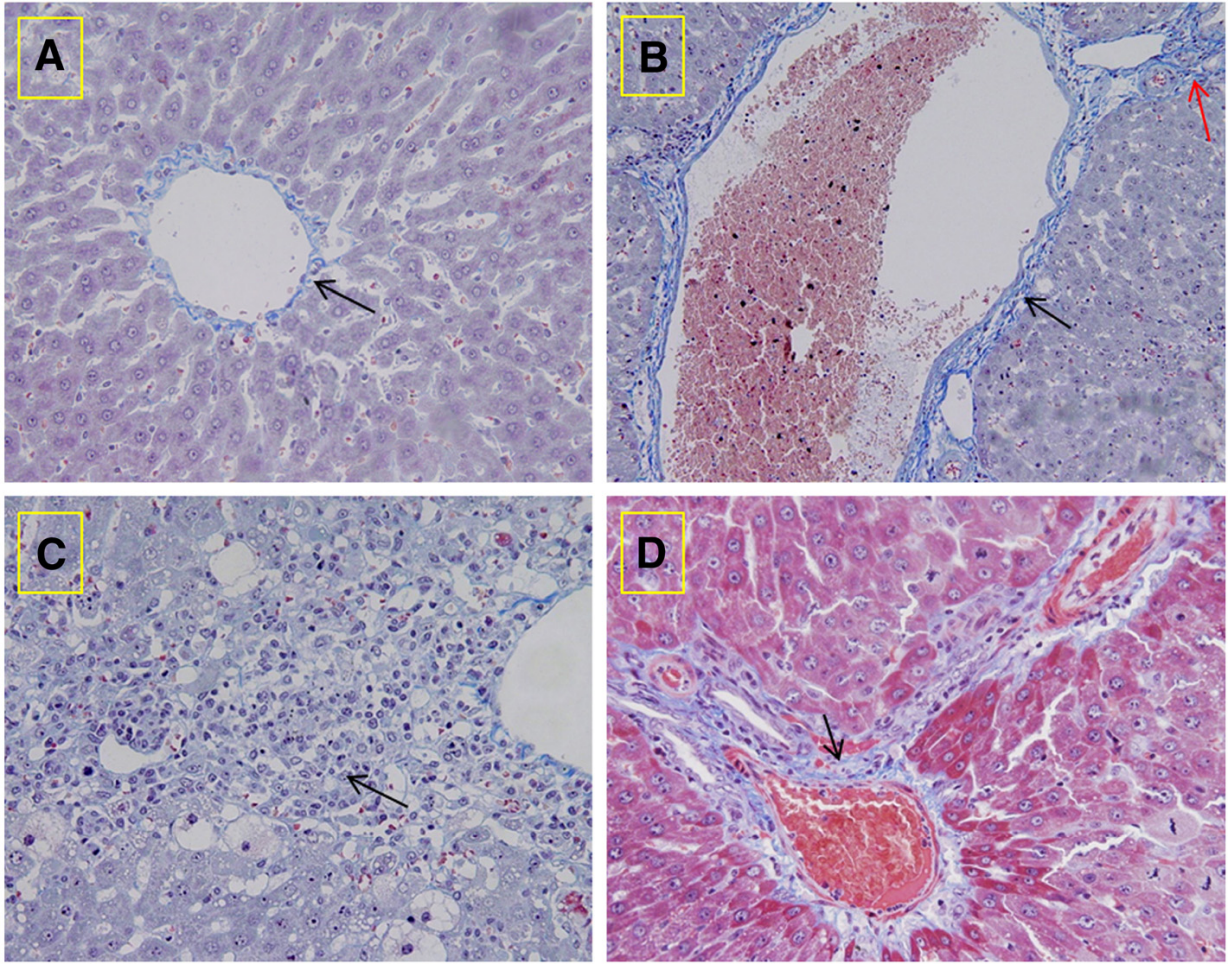
**Histological assessment of liver fibrosis in CCl4-treated rats and the effects of the combination of melatonin and folic acid on collagen deposition.** The extent of matrix deposition (arrow) was measured by Masson’s trichrome staining of liver tissue sections from control animals (**A**: 400x), CCl4-treated rats (black arrow: collagen of the very dilated central vein; red arrow: the interlobular collagen deposition) (**B**: 200x), CCl4-treated rats to show the inflammatory cells (**C**: 400x) and the rats receiving melatonin and folic acid (black arrow: collagen of the central vein) (**D**: 400x).

Histopathological changes of collagen deposition occurred in CCl4-challenged rats and prevention by the treatment with melatonin and folic acid are showed in Figure 2. The tissues of rats treated with CCl4 revealed extensive accumulation of connective tissue resulting in formation of continuous interlobular septa, noticeable alterations and dilations in the central vein and pronounced inflammation as compared to the normal control (Figure 2B, 2C). The group challenged with CCl4 and treated with melatonin and folic acid resulted in less destruction of the liver architecture without fibrosis and moderate inflammation (Figure 2D).

## Discussion

This study investigated the capability of folic acid, melatonin or the combination of these two compounds to protect against CCl4-induced hepatotoxicity. Our findings confirmed the previous observations that melatonin [28] and folic acid [29] effectively reduce oxidative stress, restore the normal concentrations of anti-oxidant enzymes, and exhibit antihyperlipidemic activity. In addition, the antioxidant activity of natural products has a number of beneficial effects in the treatment of various types of diseases [30].

The hepatotoxicity of CCl4 includes the production of free radicals and the activation of Kupffer cells and macrophages, which generate inflammatory and profibrogenic mediators. The overproduction of free radicals is the initial step in a chain of events that eventually leads to membrane lipid peroxidation and ultimately to cellular apoptosis and necrosis [31]. The potential mechanisms of chemical-induced liver apoptosis include an increase in cytokine concentrations and/or oxidative stress [32]. In addition, lipid peroxidation, a ROS-mediated mechanism, has been implicated in the pathogenesis of various liver injuries and the subsequent liver fibrogenesis that is observed in experimental animals [33].

We found that the concentration of MDA and hydroperoxide, which are indices of oxidative stress, were elevated in CCl4-challenged rats. However, decreased concentrations of these two compounds were found in the liver tissues of those CCl4-rats that were treated with either melatonin, folic acid or the combination of melatonin and folic acid, which suggests that these compounds can effectively protect against lipid peroxidation. Melatonin exerts antioxidant protection in different cell organelles, both *in vitro* and *in vivo*[34]. The body has an effective defense mechanism to prevent and neutralize damage that is induced by free radicals. These enzymes constitute a mutually supportive team of defense against ROS [35]. Melatonin and folic acid were found to significantly restore the normal concentrations of GSH and the catalase activity. The catalase enzyme, which exists in all aerobic cells, is a hemeprotein that metabolizes the decomposition of H2O2 to form oxygen and water. GSH acts as a non-enzymatic anti-oxidant that reduces the amount of H2O2, hydroperoxides and xenobiotic toxicity [36]. The significant decrease of hydroperoxide in the blood and hepatic tissues confirmed that the pre-treatment with folic acid and melatonin could effectively protect against the hepatic lipid peroxidation that is induced by CCl4. The anti-oxidant activity and antilipidemic effects of melatonin may enhance the modulation of blood pressure and most likely play the most important role in the amelioration of the damage to the target organ [37]. GSH is a crucial determinant for cell survival or death in oxidative stress conditions [38] and, thus, GSH is critical in reducing the toxic effects of CCl4. Moreover, the increase in hepatic GSH concentrations in rats treated with either folic acid, melatonin or their combination may be due to an increase in the amount of GSH synthesis or regeneration.

ROS upregulates NF-κB, which is required for the induction of pro-inflammatory cytokines, such as IL-1ß, TNF-α and IL-6 [39]. TNF-α is a key mediator of the immune and inflammatory responses and controls the expression of the inflammatory gene network. Therefore, the overproduction of TNF-α contributes significantly to the pathological complications observed in many inflammatory diseases; for example, pro-inflammatory cytokines can increase the risk of schizophrenia [40]. Similarly, hepatic injury is associated with the upregulation of TNF-α gene expression that was observed in the CCl4 group; this result is therefore in accordance with previous studies [41]. Consequently, the overproduction of TNF-α contributed to the manifestation of the systemic inflammatory response and ultimately to the development of organ failure.

We found that the upregulation of TNF-α expression was accompanied by the upregulation of the Fas genes in CCl4-induced liver injury. The Fas protein is a type I membrane receptor that belongs to the TNF-receptor superfamily. Mita et al. [42] found that the expression of FasL by macrophages plays a role in their pathogenesis.

In the present study, Fas mRNA expression was significantly upregulated in CCl4- injected rats. Similarly, Zhang et al. [43] found that the expression of Fas is increased in CCl4-induced liver fibrosis. Activation of the Fas receptor by the FasL induces apoptosis via the activation of the caspase cascade [44]. Therefore, the upregulation of both TNF-α and Fas clearly explains the hepatic tissue damage and dysfunction that was observed in the CCl4 group. Furthermore, hepatic injury in rats leads to elevations of serum AST and ALT and an increased incidence and severity of histopathological hepatic lesions. The present study revealed a significant increase in the concentrations of AST, ALT and ALP upon exposure to CCl4, which indicates considerable hepatocellular injury. In addition, an increase in serum AST and ALT concentrations by CCl4 has been attributed to hepatic structural damage because these entities are normally localized to the cytoplasm and are only released into the circulatory system after cellular damage has occurred [45].

The oxidative stability that is induced by the combination of melatonin and folic acid may mediate a downregulation of NF-κB activation, which results in the suppression of the inflammatory cascade and the low concentrations of TNF-α that were observed. Thus, the hepatic injury markers were significantly retarded in the animals that received any of these treatments. In fact, folic acid and melatonin significantly attenuated the increased concentrations of the serum liver enzymes that were induced by CCl4 and therefore led to the subsequent restoration of these to normal concentrations. The effect of folic acid and melatonin was further confirmed through histopathological examinations. It was found that pre-treatment with the combination of melatonin and folic acid had broad anti-inflammatory effects and attenuated the allergic inflammation in the CCl4- challenged rats. This amelioration of the hepatic tissues by melatonin and folic acid seemed to be mediated by the inhibition of oxidative stress and therefore the suppression of NF-κB, the key regulator of inflammatory production, which results in the decreased production of pro-inflammatory cytokines.

The Akt1 signal is critical for cell survival that is triggered by growth factors, the extracellular matrix, and other stimuli [46]. Therefore, there is impaired Akt and eNOS activation in cirrhotic livers [47]. We found a downregulation of Akt1 gene expression in CCl4-challenged rats. The concentration of Akt1 mRNA expression was markedly restored in CCl4-rats that were pre-treated with the combination of melatonin and folic acid. Previous studies have shown that Myr-Akt gene therapy can restore Akt activation and NO production in cirrhotic livers, which suggests that this therapy may be helpful in treating portal hypertension [47]. The restorative capacity of melatonin and folic acid was also confirmed in the complete restoration of the IFN- γ mRNA expression to normal concentrations. The proteins EMSY and BRAC2 repress a number of IFN-stimulated genes; however, an Akt1-dependent pathway contributes to the full activation of IFN-stimulated genes by relieving this repression [48]. Similar data establishes that Akt activity is essential for the upregulation of key IFN-α- and IFN-γ-inducible proteins, which have important functional consequences in the induction of IFN responses [49]. In addition, the retrovirus-mediated expression of activated Akt in primary T cells from CD28-deficient mice is capable of selectively restoring the production of IL-2 and IFN-γ [50]. We proved that the activation and upregulation of Akt1 was accompanied by an improvement in the biomarkers of hepatic damage, the histological architecture and liver function.

We confirmed the hypolipidemic effect of melatonin and folic acid in CCl4-treated rats, which was manifested by the low concentrations of cholesterol, triglycerides, and LDL and the increased HDL concentrations. Similar findings were also reported for other experimental models, which observed a decline in LDL and total cholesterol and an increase in HDL concentrations in animals treated with melatonin [51]. This effect may be related to the enhancement of the catabolism of cholesterol to form bile acids [51] and the inhibition of cholesterol synthesis and LDL receptor activity [52].

HDL plays an essential role in the transport of cholesterol to the liver for excretion into bile acids [53], which are cytoprotective in hepatocytes because of their ability to activate phosphatidylinositol-3-kinase and its downstream signal Akt [54]. We confirmed the upregulation of the Akt1 signal by the combination of melatonin and folic acid. The combination treatment was better able to restore the elevated concentrations of cholesterol, triglycerides and inflammatory indicators than the individual treatments. A significant increase in the arterial elasticity index, a significant improvement in glucose and lipid metabolism, and a significant increase in HDL cholesterol have also been observed in patients treated with anti-oxidants [55]. Additionally, the beneficial effect of anti-oxidants on LDL oxidation has been previously demonstrated [56].

Our findings provide evidence of the potential anti-oxidant and anti-inflammatory effects of melatonin and folic acid. This combination restored normal oxidative stress concentrations, which might inhibit NF-κB and thus downregulate TNF-α and Fas mRNA expression. In addition, melatonin and folic acid markedly upregulated the cell survival signal, Akt1, and the IFN-γ concentrations. Furthermore, the restoration of the survival signaling genes that was induced by melatonin and folic acid also resulted in significant improvements to the liver function and the histological architecture. There are currently no data on the treatment of CCl4-induced hepatic injury with melatonin and folic acid that would indicate whether this intervention has a significant clinical impact. We have elucidated a potential role for these substances in the treatment of hepatic injury that needs to be intensively investigated in future research that focuses on patient oriented outcomes.

## Abbreviations

ALT: Alanine Aminotransferase; AST: Aspartate aminotransferase; ALP: Alkaline phosphatase; Fas: Programmed cell death-receptor; LDH: Lactate dehydrogenase; MDA: Malondialdehyde; ROS: Reactive Oxygen Species; Akt1: Protein kinase B; IFN-γ: Interferon gamma; IL: Interleukin; TNF-α: Tumor necrosis factor-alpha.

## Competing interest

There was no conflict of interest for any of the authors of this paper.

## Authors’ contributions

HE designed the study, described histological changes, prepared figures, drafted the manuscript and performed the statistical analysis. SB designed the study and was responsible of the animal model, the biochemical investigations and drafted the manuscript. IA was responsible for the revision of the manuscript in the final form. AR and SE were participated biochemical investigations. All authors read and approved the final manuscript.
